# Exploring Bone Morphogenetic Protein-2 and -4 mRNA Expression and Their Receptor Assessment in a Dynamic *In Vitro* Model of Vascular Calcification

**DOI:** 10.3390/cells13242091

**Published:** 2024-12-18

**Authors:** Manuela Cabiati, Federico Vozzi, Elisa Ceccherini, Letizia Guiducci, Elisa Persiani, Ilaria Gisone, Agnese Sgalippa, Antonella Cecchettini, Silvia Del Ry

**Affiliations:** 1 Institute of Clinical Physiology IFC-CNR, Via Giuseppe Moruzzi 1, 56124 Pisa, Italy; federico.vozzi@cnr.it (F.V.); ceccherini@ifc.cnr.it (E.C.); letizia.guiducci@cnr.it (L.G.); elisapersiani@cnr.it (E.P.); ilariagisone@gmail.com (I.G.); silvia.delry@cnr.it (S.D.R.); 2 Health Science Interdisciplinary Center, Sant’Anna School of Advanced Studies, 56100 Pisa, Italy; agnese.sgalippa@santannapisa.it; 3 Department of Clinical and Experimental Medicine, University of Pisa, 56100 Pisa, Italy

**Keywords:** calcification, vascular smooth muscle cells, endothelial cells, BMP system

## Abstract

Background: Vascular calcification (VC) is a dynamic, tightly regulated process driven by cellular activity and resembling the mechanisms of bone formation, with specific molecules playing pivotal roles in its progression. We aimed to investigate the involvement of the bone morphogenic proteins (*BMP-2*, *BMP-4*, *BMPR-1a/1b*, and *BMPR-2*) system in this process. Our study used an advanced in vitro model that simulates the biological environment of the vascular wall, assessing the ability of a phosphate mixture to induce the osteoblastic switch in human coronary artery smooth muscle cells (HCASMCs). Methods: HCASMCs were grown in mono- and co-culture with human coronary artery endothelial cells (HCAECs) in a double-flow bioreactor (LiveBox2 and IVTech), allowing static and dynamic conditions through a peristaltic pump. The VC was stimulated by incubation in a calcifying medium for 7 days. A *BMP* system Real-Time PCR was performed at the end of each experiment. Results: In monocultures, *BMP-2* expression increased in calcified HCASMCs in static (*p* = 0.01) and dynamic conditions. *BMP-4* and the biological receptors were expressed in all the experimental settings, increasing mainly in dynamic flow conditions. In co-cultures, we observed a marked increase in *BMP-2* and *BMP-4*, *BMPR-1a* (*p* = 0.04 and *p* = 0.01, respectively), and *BMPR-2* (*p* = 0.001) in the calcifying setting mostly in dynamic conditions. Conclusions: The increase in *BMP-2/4* in co-culture suggests that these genes might promote the switch towards an osteogenic-like phenotype, data also supported by the rise of both *BMPR-1a* and *BMPR-2*. Thus, our findings provide insights into the mechanisms by which dynamic co-culture modulates the *BMP* system activation in an environment mimicking *in vivo* VC’s cellular and mechanical characteristics.

## 1. Introduction

Vascular calcification (VC) was once viewed as a passive process, characterised by the accumulation of calcium and phosphate in the arteries, leading to serious clinical outcomes. As such, VC is now recognised as a reliable indicator of future cardiovascular complications, including heart attacks and strokes [1,2,3,4].

Recent research has demonstrated that VC is an active, tightly regulated, cell-mediated process similar to bone formation in both its development and progression [5,6,7,8].

These processes are driven by the activation of various bone-associated proteins, calcification-promoting factors, and multiple signalling pathways in different cell types, such as vascular smooth muscle cells (VSMCs), macrophages, and endothelial cells (ECs). VSMCs, typically found in the medial layer of blood vessels where they regulate vascular tone, normally exhibit a contractile phenotype and express genes crucial for maintaining the myofilament structure and function. However, under conditions of biological stress or injury, VSMCs can transition from a quiescent, differentiated state to an actively proliferating and synthesising phenotype. In this activated state, they can spontaneously generate a calcified matrix and undergo a bone-like trans-differentiation into osteoblast- or chondrocyte-like cells, contributing to vascular calcification (VC) [6,9,10,11,12].

Recently, this process, called the bone–vascular axis theory [13,14], has provided a new paradigm for the study of VC, and many papers have reported the role of bone morphogenic proteins (*BMP*s), a group of ligand proteins with pleiotropic functions, as key molecules in the progression of VC. The *BMP* signalling pathway is pivotal for osteogenesis, a process of endothelial-to-mesenchymal transition (EndMT), and VSMC calcification [15].

The *BMP* family is part of a larger group of over thirty ligand molecules that interact with specific receptors, co-receptors, and signalling–regulating proteins, collectively known as the transforming growth factor (*TGF-β*) superfamily [16,17].

*BMP*s initiate intracellular signalling by binding to specific receptors, forming complexes that consist of type I (*ALK2*, *ALK3,* or *BMPR-1a*, and *ALK6* or *BMPR-1b*) and type II serine/threonine kinase receptors (*BMPR-2*, *ACVR2A*, and *ACVR2B*) [18]. *BMP-2* and *BMP-4* play a key role in vascular calcification (VC) by preferentially binding to *BMPR-1a* and *BMPR-1b* in combination with *BMPR-2*, promoting bone formation through these pathways [19,20]. They facilitate the recruitment of osteoblast-like and chondroblast-like cells, driving calcific remodelling and endochondral-like ossification. Beyond VSMCs, the BMP family can act on other cell types such as ECs and myofibroblasts. *BMP-2* and *BMP-4*, along with traditional osteoblast and osteoclast regulatory proteins, show distinct expression patterns in calcified atherosclerotic plaques [21,22].

This observation led to the suggestion that *BMP*s act on VC in the same manner as they do in normal bone formation [17].

The activation of both the BMP and TGF-β families is pivotal in embryonic development; they maintain balance between the osteoclast and osteoblast activity in adults, and contribute to the onset of numerous diseases. The highly conserved core of the canonical TGF-β/*BMP* signalling is a simple linear cascade that includes *BMPR-1a*, *BMPR-1b,* and *BMPR-2* controlling several events, as well as cell proliferation, differentiation, apoptosis, migration, extracellular matrix (ECM) remodelling, immune functions, and tumour invasion/metastasis [23,24].

This study aimed to assess the expression of BMP system components (*BMP-2*, *BMP-4*, *BMPR-1a/1b*, and *BMPR-2*) in an advanced in vitro model designed to mimic the biological environment of the vascular wall. This evaluation involved examining the ability of a mixture of phosphates to induce the initial phases of the osteoblastic transformation of human coronary artery smooth muscle cells (HCASMCs), a critical event in VC. The model allowed the indirect contact of the HCASMC and EC co-culture under static and dynamic flow conditions.

## 2. Materials and Methods

### 2.1. Cell Cultures

Primary human coronary artery endothelial cells (HCAECs) and HCASMCs (both from Lonza, Milan, Italy) were used in this study at a seeding density of 15,000 cells/cm². HCAECs were cultured in Endothelial Cell GM MW2 growth medium, supplemented with the necessary growth factors and Penicillin/Streptomycin at final concentrations of 100 I.U./mL and 100 μg/mL, respectively. HCASMCs were maintained in Smooth Muscle Medium, which comprised Medium 231 supplemented with Smooth Muscle Growth Supplement (Lonza, Milan, Italy) and Penicillin/Streptomycin at the same final concentrations (Reference medium). Both cell types were incubated at 37 °C with 5% CO_2_, and the culture media were refreshed every 3 to 4 days until the cells were prepared for use in the bioreactor [25,26].

#### 2.1.1. Dynamic and Static Device Experimental Setting

As previously reported, HCAECs and HCASMCs were cultured using the LiveBox2 (LB2) dual-flow bioreactor system (IVTech Srl, Pisa, Italy). This system features two independently perfusable chambers separated by a PET membrane with circular pores of 45 μm diameter [25,26]. Each upper chamber was seeded with 45,000 endothelial cells (ECs) and maintained in the endothelial growth medium, while the lower chambers were seeded with 30,000 HCASMCs and cultured in reference medium (Medium 231 supplemented with Smooth Muscle Growth Supplement, Lonza). Cells originated from two different donor batches and were used in three independent experiments.

The upper chambers were connected to a peristaltic pump, enabling a dynamic flow of 250 µL/min, while the static condition was maintained without fluid circulation. To promote calcification in the HCASMCs, the reference medium was replaced with a calcifying medium comprising 1.9 mM NaH_2_PO^4^/Na_2_HPO^4^ (1:1) in high-glucose DMEM.

The LB2 bioreactor was incubated at 37 °C with 5% CO_2_ in a humidified cell culture incubator for 7 days, with the medium being refreshed after 72 h [25,26]. HCAECs are used to release growth factors, creating a growth environment similar to physiological conditions, but ECs are not used for molecular analysis. By the term “co-culture” we refer only to HCASMCs grown with the factors released by the ECs for 7 days.

#### 2.1.2. Cell Viability

Cell viability was assessed after the 7-day experiment using the CellTiter-Blue^®^ Cell Viability Assay kit (Promega, Milan, Italy). This assay relies on the ability of metabolically active cells to reduce resazurin to resorufin, a fluorescent compound measurable for quantification.

Briefly, the culture medium contained in the upper and lower chambers was removed and replaced with 1 ml of CellTiter-Blue^®^ solution at 10% *v*/*v*, obtained by mixing endothelial medium or reference medium with CellTiter-Blue^®^ reagent, respectively. Following 2.5 h of incubation, 100 μL of solution was collected from each chamber, and the fluorescence at 560/590 nm was recorded using the FLUOstar Omega microplate reader (BMG Labtech, Ortenberg, Germany). At the end of the viability assay, the cells were washed 3 times with PBS without calcium and magnesium. HCAECs were stored at −80 °C and HCASMCs were subjected to intracellular calcium determination.

#### 2.1.3. Intracellular Calcium Assay

HCASMC calcification was assessed using the Calcium Colorimetric Assay Kit (Sigma-Aldrich, Milan, Italy), following a previously described protocol [26]. In summary, cells were lysed with 0.6 M HCl for 1 h at 4 °C, followed by incubation overnight at −20 °C. The absorbance was then measured at 575 nm using a FLUOstar Omega microplate reader (BMG Labtech, Ortenberg, Germany).

### 2.2. RNA Extraction and Real-Time PCR Experiments

Total RNA was extracted from HCASMCs (reference and calcifying under static condition, n = 9 samples for each condition; and reference and calcifying under dynamic condition, n = 9 samples for each condition) and co-cultures (HCASMC growth in presence of HCAEC in the upper chamber of the LB2; reference and calcifying under static condition, n = 9 samples for each condition; and reference and calcifying under dynamic condition, n = 9 samples for each condition) using the RNeasy Plus Micro Kit (Qiagen SpA, Milano, Italy), which is specifically designed to purify RNA from small cell quantities (<5 × 10^5^ cell number), as detailed in our previous studies [26,27].

In summary, cells were resuspended and lysed in a guanidine isothiocyanate-based denaturing buffer to rapidly inactivate RNases and preserve RNA integrity. The lysates were processed through a gDNA Eliminator spin column to remove genomic DNA. Ethanol was then added to the flow-through to optimise RNA-binding conditions. The samples were transferred to a silica membrane (RNeasy MinElute spin column) and centrifuged at 12,000 RPM for 30 s. Specific buffers facilitated RNA binding to the membrane while thoroughly washing away contaminants. The purified RNA was eluted in RNase-free water, eliminating the need for additional DNase treatment.

The RNA concentration was quantified by measuring absorbance at 260 and 280 nm using a NanoDrop spectrophotometer (ThermoFisher, Waltham, MA, USA), with expected A260/A280 ratios between 1.8 and 2.1. Calculations followed the Beer-Lambert law.

The total RNA was reverse-transcribed into first-strand cDNA using the miScript RTII Synthesis Kit (Qiagen). Gene expression analysis was performed by Real-Time PCR on a Bio-Rad C1000 TM thermal cycler (CFX-96 Real-Time PCR Detection System, Bio-Rad) with EvaGreen dye (SsoFAST EvaGreen Supermix, Bio-Rad) for fluorescence detection. Primers for both target and reference genes were designed using Beacon Designer^®^ software (version 8.1; Premier Biosoft International, Palo Alto, CA, USA) based on GenBank sequences from the NCBI database (http://www.ncbi.nlm.nih.gov/Genbank/index.html accessed on 2 September 2024). The specificity of PCR products was confirmed by melting curve analysis, conducted by incrementally increasing the temperature from 65 °C to 95 °C in 0.5 °C steps per cycle.

The experiment adhered to the MIQE Guidelines [28] to ensure accuracy and reproducibility (Table 1).

### 2.3. Statistical Data Analysis

Cell viability data were reported as mean ± SD, with statistical significance defined as a *p*-value < 0.05. All experiments were performed independently at least three times for verification, and data analysis was conducted using Prism 8 software (GraphPad Software). Two reference genes were evaluated, and their stability was assessed using GeNorm analysis, integrated into Bio-Rad’s CFX96 Manager software (CFX-96 Real-Time PCR Detection System, Bio-Rad Laboratories Inc., Hercules, CA, USA), to identify the most consistently expressed genes.

The statistical analysis of the results was carried out through the Stat-View 5.0.1 software released for Windows Statistical (1992-98, SAS Institute Inc., SAS Campus Drive, Cary, NC, USA). The mRNA expression data were normalised by the geometric mean of the most stably expressed genes (*eEF1a* and *RNSP1*), and the relative quantification was performed by the ∆∆Ct method. Results were expressed as mean ± SEM. For the multiple comparisons, a Kruskal–Wallis non-parametric test followed by pairwise comparisons performed applying Dunn’s post hoc test with a Bonferroni correction was carried out. The equations of linear correlations were obtained using the least-squares method.

## 3. Results

### 3.1. Mono- and Co-Culture Characterisation

#### 3.1.1. Viability

At the end of each experiment, HCASMCs viability was investigated. As expected, in the HCASMC the calcifying medium reduced the cell viability in dynamic condition (*p* = 0.0003), confirming the results previously observed in static [26]. Surprisingly, the presence of HCAECs contributed to the reduction in HCASMC viability in the static model (*p* <0.0001) as yet detected in the dynamic one (*p* < 0.0001) [26].

#### 3.1.2. Evaluation of the Calcification Process

The intracellular calcium amount was quantitatively measured following 7 days of HCASMC treatment. In Table 2 are reported the concentration obtained in the experimental setting analyzed.

The results obtained highlighted a significant increase in intracellular calcium amount in calcifying condition compared to reference in monocultures both under static and dynamic condition. In co-cultures under static conditions, the intracellular calcium levels exhibited a trend consistent with that observed in HCASMCs. As reported in our previous study [26], co-cultures under dynamic conditions also demonstrated a significant increase in intracellular calcium levels (*p* = 0.003).

#### 3.1.3. Evaluation of the Switch to Osteogenic-like Phenotype

**Figure 1:**
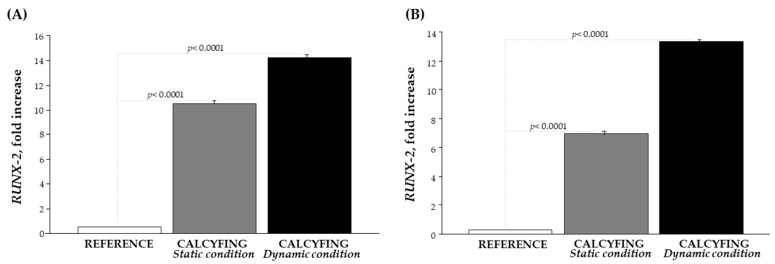
Expression of *RUNX-2* in calcifying environment: (**A**) *RUNX-2* fold increase in HCASMC subjected to static (dark grey bar) and dynamic conditions (black bar) compared with reference; and (**B**) *RUNX-2* fold increase in the HCASMC co-culture subjected to static (dark grey bar) and dynamic conditions (black bar) compared with reference.

RUNX-2 is a key regulator of vascular smooth muscle cell osteogenic transition. As expected we found a significantly marked increased expression of RUNX-2 associated with HCASMC calcification, both in monocultures than in co-cultures in presence of a calcifying environment, supporting its role in VC. In particular, RUNX-2 mRNA levels increased nearly 30-fold in HCASMCs exposed to calcifying medium under static conditions compared to the reference, and by more than 50-fold under dynamic conditions (Figure 1). In HCASMC co-cultures RUNX-2 has the same behaviour increasing nearly 30-fold in dynamic condition (Figure 1) compared with reference.

### 3.2. Real-Time PCR Analysis

#### 3.2.1. Expression Level of BMP System in HCASMC Under Static and Dynamic Conditions

**Figure 2:**
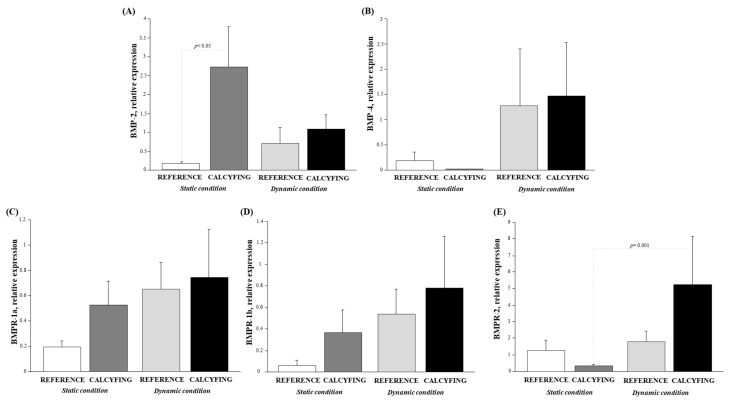
Relative mRNA expression levels of (**A**) *BMP-2*, (**B**) *BMP-4*, (**C**) *BMPR-1a*, (**D**) *BMPR-1b*, and (**E**) *BMPR-2* in HCASMC in the absence/presence of calcifying environment and subjected to static (white and dark grey bars) and dynamic conditions (light grey and black bars).

The expression levels of *BMP-2* and *BMP-4* and their specific membrane receptors *BMPR-1a*, *BMPR-1b*, and *BMPR-2* in HCASMC subjected to static or dynamic flow conditions in the absence/presence of phosphate-induced calcification are shown in Figure 2.

In both static and dynamic flow conditions, *BMP-2* (Figure 2) showed a marked increase in the calcifying environment with respect to control cells, although this was significant only in static conditions (*p* < 0.05).

The *BMP-4* mRNA expression increased in dynamic conditions with respect to static conditions, where it was lower in the presence of the calcifying medium, albeit not significantly. A slight trend towards increased HCASMC calcification was observed during dynamic flow, as depicted in Figure 2 This suggests that the effect of flow on *BMP-4* expression levels is more pronounced than the influence of calcification.

Regarding the BMP receptors (Figure 2), it was observed that they were expressed in all the experimental settings. mRNA levels were higher under dynamic flow conditions compared to static ones, except for *BMPR-2* in the static setting, where its expression in the presence of calcification appeared slightly lower, although not significantly. This could indicate a compensatory mechanism to counterbalance the biological effects of *BMP-2*. As to *BMP-2*, both *BMPR-1a* and *BMPR-1b* appear to be influenced by the calcifying environment rather than the flow. In contrast, *BMPR-2* seems to be modulated by dynamic flow as its specific ligand *BMP-4*.

Table 3 reports the correlations between *BMP-2* and *BMP-4* and their specific receptors, in static (Table 3a) and dynamic (Table 3b) conditions, confirming that BMP signalling is mediated directly through the interaction with these receptors.

**Figure 3:**
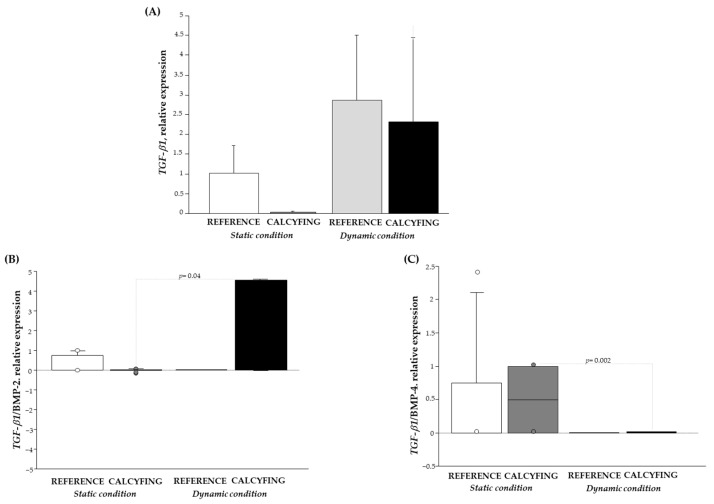
Transcript levels: (**A**) relative mRNA expression levels of *TGF-β1* in the absence/presence of calcifying environment and subjected to static (white and dark grey bars) and dynamic conditions (light grey and black bars) [Fisher’ test: *p* = 0.03; Bonferroni/Dunn’s post hoc test: *p* = ns calcifying medium static vs. dynamic]; and (**B**) mRNA expression ratio of *TGF-β1/BMP-2* and (**C**) *TGF-β1/BMP-4* in HCASMC expressed as box plots in the absence/presence of calcifying environment and subjected to static (white and dark grey plots) and dynamic conditions (light grey and black plots). Each box consists of five horizontal lines displaying the 10th, 25th, 50th, 75th and 90th percentiles of the variable. All values above the 90th percentile and below the 10th percentile, are plotted separately and represents the outliers.

Examining the mRNA expression of *TGF-β1* (Figure 3A), an additional member of the BMP family, a decrease in its expression level in HCSMC calcification under static and dynamic conditions was revealed, indicating that *TGF-β1* is influenced by flow. When considering the ratio of *TGF-β1/BMP* members, a notable increase in the *TGF-β1/BMP-2* expression ratio is observed within the calcifying environment under dynamic conditions, contrasting with a significant decrease in the same experimental setting in static conditions (Figure 3B). Instead, the trend showcased by the *TGF-β1/BMP-4* expression ratio was the opposite (Figure 3C), highlighting the role of these two complexes in the development of atherosclerosis [29,30,31].

#### 3.2.2. Expression Level of BMP System in Co-Cultures Under Static and Dynamic Conditions

**Figure 4:**
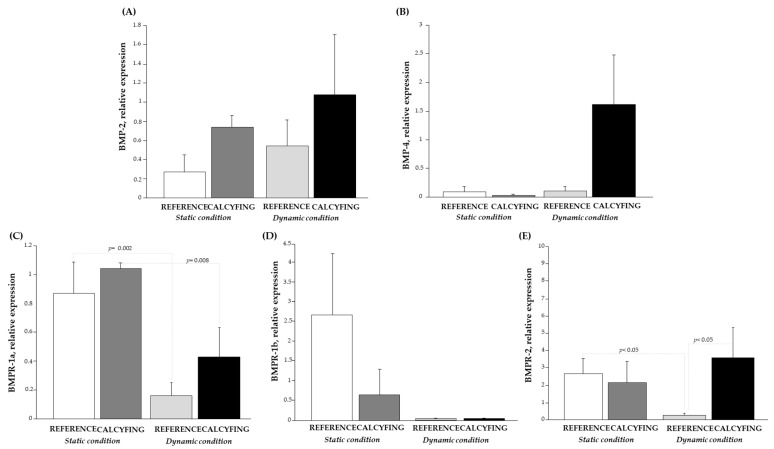
Relative mRNA expression levels of (**A***) BMP-2*, (**B**) *BMP-4*, (**C**) *BMPR-1a*, (**D**) *BMPR-1b*, and (**E**) *BMPR-2* in the HCASMC co-culture system in the absence/presence of a calcifying environment and subjected to static (white and dark grey bars) and dynamic conditions (light grey and black bars).

The co-culture experimental settings, in both static/dynamic conditions with or without calcifying medium, and completed with the presence of HCAECs (indirect co-cultures) allowed us to extrapolate the effect of the endothelial cells on the *BMP* system. As reported in Figure 4, in the co-cultures, we observed a marked increase in *BMP-2* (Figure 4) in the calcifying setting with respect to control cells, both in static and dynamic conditions, albeit not significant, indicating a lack of HCAEC effect. At the same time, *BMP-4* (Figure 4) had a higher result in the presence of the calcifying medium in dynamic conditions (Fisher’ test: *p* = 0.04; Bonferroni/Dunn’s post hoc test: *p* = ns).

A counter-regulation was found between the *BMPR-1a* and the other receptors, particularly in static conditions (Figure 4C–E). In dynamic and calcifying conditions, higher levels were observed for *BMPR-1a* (Fisher’ test: *p* = 0.01; Bonferroni/Dunn’s post hoc test: *p* = ns) and *BMPR-2* (Fisher’ test: *p* = 0.001; Bonferroni/Dunn’s post hoc test: *p* < 0.05), while the *BMPR-1b* mRNA levels remained in a steady state in a dynamic environment. These results evidenced that HCAEC seems to affect mainly the relative expression of BMPR-1a and *BMPR-1b* receptors.

Significant correlations were observed between the members of the *BMP* family only in dynamic conditions (Table 4). This coordinated regulation ensures that the BMP signalling intensity and effects are appropriately matched to the VC.

Moreover, in co-culture, the mRNA profile trend of *TGF-β1* was similar to those observed in HCASMCs, decreasing in the calcifying environment in the static condition (control: 2.12 ± 1.27; calcifying: 1.08 ± 0.76) and remaining in a steady state in the dynamic one (control: 2.86 ± 1.66; calcifying: 2.31 ± 2.13). The analysis of the *TGF-β1/BMP-2* and *TGF-β1/BMP-4* ratio in the co-culture experimental setting evidences the increase in *TGF-β1/BMP-2* in the presence of the calcifying environment both in the static condition (0.502 ± 0.49 vs. 1.93 ± 1.06, HCASMC co-culture vs. HCASMC co-culture with calcifying medium) and in the dynamic condition (1.02 ± 0.01 vs. 6.05 ± 2.47 HCASMC co-culture vs. HCASMC co-culture with the calcifying medium), even though not statistically significant.

## 4. Discussion

In the past two decades, extensive research has demonstrated that VC is a closely regulated process, exhibiting many similarities to bone mineralisation. This includes the need for the coordinated activation of *BMP/TGF-β1* family members, which play a crucial role in the development of calcific cardiovascular disease [17].

In this study, we used an advanced dynamic *in vitro* co-culture system using endothelial and smooth muscle cells to replicate vascular tissue complexity and shed light on the pathophysiology of VC [25,26].

We have investigated the *BMP-2* and *BMP-4* systems as composed of important mechanosensitive molecules able to modulate vascular endothelial proliferation in response to dynamic flow. These molecules exert pro-inflammatory and pro-atherogenic effects, promoting oxidative stress, endothelial dysfunction, and osteogenic differentiation [17,21].

Our results are consistent with the literature, showing that HCASMCs cultured in high phosphate levels switch from a contractile to an osteoblast-like phenotype, leading to vascular calcification [4,32,33,34,35,36], data also confirmed by the marked increase in the *RUNX-2* expression.

Briefly, *BMP-2*, *BMP-4*, *BMPR-1* and *BMPR-2* were found to be differentially expressed in the calcifying environment both in static and in dynamic conditions. In particular, the analysis of HCASMC monocultures under standard (static) and dynamic conditions shows that, as expected, *BMP-2* expression increases in the calcifying medium; however, the flow moderates this increase.

Moreover, as also demonstrated in a previous study of ours [27], the results obtained on *BMP-4* mRNA expression, allowed us to confirm the literature’s findings which suggest that *BMP-4* levels are lower under physiological conditions compared to pathological ones, in the absence of flow or medical devices used to prevent atherosclerosis, identifying the flow as the most feasible appropriate for the study of this marker [27,37].

Conversely, the expression of *BMP-4* and *BMPR-2* does not increase in standard static culture conditions; their expression decreases in the presence of the calcifying medium. However, they show a significant increase in dynamic cultures, even in the control group, indicating a noteworthy impact of flow on the expression of these markers. As far as *BMPR-1a* and *BMPR-1b* are concerned, they increase with the calcifying medium, but there is no modulation due to the flow.

It well-known that the expression of *BMPR-2*, the receptor for classic osteogenic ligands *BMP-2* and *BMP-4*, is decreased in patients with advanced coronary atherosclerosis and it is protective against atherosclerosis in animal models [38], suggesting a context-specific role of BMPR-2-mediated signalling. Here, we observed a marked increase in *BMPR-2* in HCASMCs under dynamic conditions. The expression of *BMPR-1a*, *BMPR-1b*, and *BMPR-2* act as a bridge to promote VC playing a crucial role in the transdifferentiation of VSMCs into the osteoblast or chondrocyte-like phenotype. A plethora of literature indicates that ECs play a significant role in VC via mechanotransduction [39,40,41]. The increase in both *BMPR-1a* and *BMPR-2* might underline the role of these receptors in inducing the VSMC switch to the osteogenic-like phenotype, further promoting VC. The increase in the *TGF-β/BMPs* ratio also drives this mechanism [42]. This phenomenon in our model is more marked in static conditions, but it is important to underline it under dynamic conditions, which better simulate the *in vivo* environment. Considering the flow variable, probably more days of co-cultures are necessary to better mark this increase, but taken together, our data indicate that the *BMP-2* and *-4* systems play environmental, tissue-specific, and time-dependent roles in the activation of the VC process.

In summary, we could hypothesise the following:

1. When comparing a monoculture to co-culture, we observe an increase in *BMP-2* mRNA expression in a calcifying environment under dynamic conditions (flow). This suggests that the flow, rather than the presence of HCAECs, is likely driving this trend.
2. Comparing a monoculture and co-culture shows that both flow and the presence of HCAECs modulate *BMP-4* expression. However, the *BMP-4* expression is not affected by the calcifying medium, as its pattern remains similar under static conditions, with a general tendency to decrease.
3. *BMPR-1a* and *BMPR-1b* transcript levels are modulated by a phosphate mixture, similar to *BMP-2*, without any apparent influence from HCAECs.
4. The *BMPR-2* expression is impacted by flow but not by HCAECs.

## 5. Conclusions

In conclusions, the study highlights the involvement of the bone morphogenetic-related protein members (*BMP-2*, *BMP-4* and their biological receptors) in VC and emphasises the significance of pro and anti-calcifying mechanisms in VC pathophysiology. Although additional studies on strategies for suppressing the action of the BMP system may offer clinical benefits for preventing or treating VC, our results could provide a starting point to better understand a possible mechanism by which alterations in BMP signaling play a role in VC pathogenesis by affecting the osteogenic differentiation of vascular smooth muscle cells. Moreover, the study provides insights into how dynamic co-culture modulates the activation of the BMP system in an environment mimicking VC’s cellular and mechanical characteristic *in vivo*. Further investigations of the spatiotemporal expression and activation of the BMP/TGF-β family are needed to define how these markers are integrated and eventually to design therapeutic interventions beneficial in specific conditions.

## 6. Study Limitation

The main limitations of the are as follows: the small number of samples analysed due to the challenging bioreactor to use the lacking of protein assay and experiments of knock down gene expression evaluations.

The *in vitro* system was designed for evaluation from a biomolecular perspective only, through Real-Time PCR studies of cell-specific parameters. Indeed, this study is part of a broader project aimed at investigating the modulation of vascular calcification through the use of the LiveBox2 double-flow bioreactor (IVTech Srl, Pisa, Italy) in dynamic co-cultures of HCAEC and HCASMC mimicking human physiology.

## Figures and Tables

**Table 1:** Primer sequence details of the analysed gene.

GENE	PRIMER SEQUENCE, 5′ → 3′	GENEBANK	AMPLICON LENGHT, bp	LOCATION	Ta	EFFICIENCY	R2
***RNSP1***	**F:** ACCCATGGTAGTTGCTGCTC **R:** AGCTGGCTCTCCACTCACTC	NM_006711.3	103	16p13.3	60 °C	97.3%	0.999
***eEF1a***	**F:** CTTTGGGTCGCTTTGCTGTT **R:** CCGTTCTTCCACCACTGATT	NM_001402	183	6q13	60 °C	101.7%	0.998
***RUNX-2***	**F:** GATTCTTAACCAACCAGCCTTACC **R:** AGTGATGTCATTCTGCTCCTCTAA	NM_001024630.4	120	6p21.1	60 °C	96.4%	0.994
***BMP2***	**F:** CGTCAAGCCAAACACAAACAG **R:** AGCCACAATCCAGTCATTCC	NM_001200.4	105	20p12.3	58 °C	98.5%	0.997
***BMP4***	**F:** TGGAATGACTGGATTGTG **R:** ATGGTTGGTTGAGTTGAG	NM_001202	99	14q22.2	60 °C	103.5%	0.990
***BMPR-1a***	**F:** CGAAGATATGCGTGAGGTTGT **R:** AGTCTGGAGGCTGGATTGT	NM_004329	135	10q23.2	58 °C	95.8%	0.992
***BMPR-1b***	**F:** GGACATAGAACGGAACTCAT **R:** GCATTCACATTACCATAGCG	NM_001203	106	4q22.3	58 °C	100.5%	0.998
***BMPR-2***	**F:** TGATGGAACCTGTGTTATTAGTGA **R:** GATAGTGCCAACCTCGCTTA	NM_001204	112	2q33.1-2	60 °C	98.7%	0.995
***TGF-β1***	**F:** TGAACCCGTGTTGCTCTC **R:** GCCAGGAATTGTTGCTGTATT	NM_000660.7	104	19q13.2	60 °C	104.9%	0.991

**Table Legend. *RNSP1*:** RNA-binding protein with serine rich domain 1; ***eEF1a*:** Eukaryotic translation elongation factor 1 alpha 1; ***RUNX-2:*** RUNX family transcription factor 2; ***BMP-2*:** bone morphogenetic protein 2; ***BMP-4*:** bone morphogenetic protein 4; ***BMPR-1a*:** bone morphogenetic protein types I receptor a; ***BMPR-1b*:** bone morphogenetic protein types I receptor b; ***BMPR-2*:** bone morphogenetic protein types II receptor; ***TGF-β1***: transforming growth factor beta 1.

**Table 2:** Evaluation of the calcification process: intracellular calcium amount quantification.

EXPERIMENTAL SETTING		REFERENCE MEDIUM	CALCIFYING MEDIUM	*p*
		*Intracellular Calcium Amount*	*Intracellular Calcium Amount*	
Monoculture	**Static condition**	7.0 × 10^−3^ (μg/cell) × 10^4^	5.16 × 10^−1^ (μg/cell) × 10^4^	<0.0001
	**Dynamic condition**	4.8 × 10^−2^ (μg/cell) × 10^4^	8.7 × 10^−2^ (μg/cell) × 10^4^	0.058
Co-culture	**Static condition**	2.0 × 10^−3^ (μg/cell) × 10^4^	5.3 × 10^−2^ (μg/cell) × 10^4^	0.003

**Table 3:** (**a**) correlations in HCASMC among BMP family members in static conditions. (**b**) correlations in HCASMC among BMP family members in dynamic conditions.

(**a**)					
	*BMP-2*	*BMP-4*	*BMPR-1a*	*BMPR-1b*	*BMPR-2*
*BMP-2*	-	ns	ns	ns	R^2^ = 0.31; *p* = 0.04
*BMP-4*	ns	-	R^2^ = 0.66; *p* = 0.0002	ns	R^2^ = 0.88; *p* < 0.0001
*BMPR-1a*	ns	R^2^ = 0.66; *p* = 0.0002	-	ns	ns
*BMPR-1b*	ns	ns	ns	-	ns
*BMPR-2*	R^2^ = 0.31; *p* = 0.04	R^2^ = 0.88; *p* < 0.0001	ns	ns	-
(**b**)					
	*BMP-2*	*BMP-4*	*BMPR-1a*	*BMPR-1b*	*BMPR-2*
*BMP-2*	-	R^2^ = 0.45; *p* = 0.03	ns	ns	ns
*BMP-4*	R^2^ = 0.45; *p* = 0.03	-	R^2^ = 0.91; *p* < 0.0001	R^2^ = 0.44; *p* = 0.05	ns
*BMPR-1a*	ns	R^2^ = 0.91; *p* < 0.0001	-	ns	R^2^ = 0.60; *p* = 0.009
*BMPR-1b*	ns	R^2^ = 0.44; *p* = 0.05	ns	-	R^2^ = 0.60; *p* = 0.01
*BMPR-2*	ns	ns	R^2^ = 0.60; *p* = 0.009	R^2^ = 0.60; *p* = 0.01	-

**Table 4:** Correlations in HCASMC co-culture among BMP family members in dynamic conditions.

	*BMP-2*	*BMP-4*	*BMPR-1a*	*BMPR-1b*	*BMPR-2*
*BMP-2*	-	R^2^ = 0.66; *p* = 0.0007	R^2^ = 0.75; *p* < 0.0001	ns	R^2^ = 0.84; *p* < 0.0001
*BMP-4*	R^2^ = 0.66; *p*= 0.0007	-	R^2^ = 0.83; *p* < 0.0001	ns	R^2^ = 0.36; *p* = 0.02
*BMPR-1a*	R^2^ = 0.75; *p* < 0.0001	R^2^ = 0.83; *p* < 0.0001	-	ns	R^2^ = 0.35; *p* = 0.02
*BMPR-1b*	ns	ns	ns	-	ns
*BMPR-2*	R^2^ = 0.84; *p* < 0.0001	R^2^ = 0.36; *p* = 0.02	R^2^ = 0.35; *p* = 0.02	ns	-

## Data Availability

The original contributions presented in the study are included in the article; further inquiries can be directed to the corresponding author.
